# Whiskey Work

**Published:** 1871-06

**Authors:** 


### WHISKEY WORK.

One of the most noted of modern writers for the press has brought himself to such a condition by the large use of liquors as to make himself too disgusting to be invited to the houses of his best friends. In his drunken fits he becomes demoniacal, outrages all the proprieties of life, and in the prime of manhood becomes a wreck, a ruin, and a disgrace to his kind. However temperate the reader of these lines may be to-day, let him bear it in everlasting remembrance, that the very first glass of wine he takes makes it possible, in spite of talent, and position, and moral principle, and religious restraint, that he shall die a drivelling drunkard, like Robert Burns, and Charles Lamb, and Edgar A. Poe, and many others greater than these.

